# Supplementary material for: Exploring virtual funding committee practices in the allocation of National Institute for Health and Care Research funding: A netnographic study
Source: F1000Res. 2024 Jul 9;13:338. Originally published 2024 Apr 23. [Version 2] doi: 10.12688/f1000research.145582.2 (PMC11193083; doi:10.12688/f1000research.145582.2)
Supplement: Peer review history

# Reviewer report: Reviewer response for version 1

**DOI:** 10.5256/f1000research.159553.r284407

**Published:** 2024-06-27

**Authors:** Becky Ioppolo

**Affiliations:** 1 University of Cambridge, Cambridge, England, UK

Blatch-Jones et al. investigate how the use of virtual platforms for funding committees in NIHR during a COVID-19 pandemic impacted the members of those committees, with implicit comparisons to face-to-face meetings, which were used previously. Through interviews, an online survey and observations of recorded meetings, the research team identified how committee members and funder staff experienced virtual meetings and the consequences the virtual format had on the meeting’s effectiveness and personal aspects. This is an interesting paper which is well written and easy to follow. I’m surprised there isn’t more research on the social / interpersonal dimension of funding committee members.

 The introduction could have benefitted from more explicit context about the importance of work like this by making mentions of bias etc. to underscore the implications of human fallibility on decision making about scientific / “objective” concerns. When the people involved in the funding committees are not fully engaged or comfortable, it theoretically could lead to different funding outcomes, which is why this work is important.

 I felt there could have been better separation between considerations that are relevant to virtual versus face-to-face and considerations that are relevant in both virtual and face-to-face, especially in Table 2. Also, you mention “The amount and level of prior preparation for virtual committee meetings was frequently mentioned by NIHR staff and all eventualities were considered to try and prevent any delays during the actual committee meeting.” But how much does this level of preparation differ from face-to-face meetings? You had also mentioned that the voting software was used in both meeting formats, so what is the net effect of moving to virtual for NIHR staff?

 Clarifications:

- Were/are the recordings publicly available or not? You say: “The virtual funding committees were recorded on the online platform used by NIHR staff to enable and assist in the minutes of the meeting outcomes which are made publicly available on the NIHR website.” And “The online funding committee meeting recordings were deleted once they were analysed.”
- It was unclear what your assumed response rate for the survey would be: “We aimed to receive a range of between 160 to 240 responses based on the average size of 20 committee members per research programme.“ Was this your aim or was this the maximum number of possible respondents? 20* how many programmes?
- Table 1: Will you explain what some of the barriers respondents considered might be? Or the scale of those barriers? Simply asking ‘are there barriers’ suggests that the answer is yes (because it is difficult to assert the complete absence of any barriers), but how intense are those barriers and what is the net effect with the positives?
- I felt that the authors could expand upon why or how virtual meetings are “less free flowing and more structured” or face-to-face meetings less so. You say it’s clear they are conducted differently but don’t explain why. In face-to-face meetings to people talk over each other more? Have more whispered asides between participants? Why isn’t/shouldn’t a face-to-face meeting be “structured”?

Typos / quick fixes

- The image resolution in Figure 1 and Figure 2 is poor making the text appear fuzzy and difficult to read.
- Either the authors have introduced a typo into the quote, “Some people do not like the longet silences and find it hard to cope with,” or the quote could benefit from a [sic].

Is the work clearly and accurately presented and does it cite the current literature?

Yes

If applicable, is the statistical analysis and its interpretation appropriate?

Not applicable

Are all the source data underlying the results available to ensure full reproducibility?

No

Is the study design appropriate and is the work technically sound?

Yes

Are the conclusions drawn adequately supported by the results?

Yes

Are sufficient details of methods and analysis provided to allow replication by others?

Yes

Reviewer Expertise:

research on research, science policy, evaluation, science funding, qualitative research

I confirm that I have read this submission and believe that I have an appropriate level of expertise to confirm that it is of an acceptable scientific standard.

# Response

**Published:** 2024-07-02

**Authors:** Amanda Blatch-Jones

**Affiliations:** Faculty of Medicine, University of Southampton, Southampton, Hampshire, UK

The introduction could have benefitted from more explicit context about the importance of work like this by making mentions of bias etc. to underscore the implications of human fallibility on decision making about scientific / “objective” concerns. When the people involved in the funding committees are not fully engaged or comfortable, it theoretically could lead to different funding outcomes, which is why this work is important.

**Thank you for your comment. Although we have mentioned bias from previous research in the introduction, we have also amended the last section under the virtual interaction section of the introduction to pick up on your point about bias.**

 I felt there could have been better separation between considerations that are relevant to virtual versus face-to-face and considerations that are relevant in both virtual and face-to-face, especially in Table 2. Also, you mention “The amount and level of prior preparation for virtual committee meetings was frequently mentioned by NIHR staff and all eventualities were considered to try and prevent any delays during the actual committee meeting.” But how much does this level of preparation differ from face-to-face meetings? You had also mentioned that the voting software was used in both meeting formats, so what is the net effect of moving to virtual for NIHR staff?

**The objective of the study was not to compare face-to-face and virtual funding committees. As we had not observed face-to-face committees it would have been unfair to try and make comparisons without first observing face-to-face meetings. We have, where possible, through the survey, asked participants about their experience of face-to-face Vs virtual meetings to offer this viewpoint.**

 Table 2 was particularly focused on considerations of virtual funding committees in the future rather than comparing to face-to-face for the reason stated above. However, this is certainly something for consideration in the future (comparing face-to-face to virtual committees).

**We have amended the text under ‘the importance of preparation for virtual committee meetings’ to take account of your comment.** **Although we were aware that committee members were used to the voting software through the interviews and survey, we did not observe face-to-face meetings. Therefore, it would be unfair to make a comparison based on virtual observations alone. However, we have added a sentence to reflect your comment.**

 Were/are the recordings publicly available or not? You say: “The virtual funding committees were recorded on the online platform used by NIHR staff to enable and assist in the minutes of the meeting outcomes which are made publicly available on the NIHR website.” And “The online funding committee meeting recordings were deleted once they were analysed.”

**The recordings are not publicly available. The NIHR staff recorded the committee meetings to assist them in the minutes of the meeting, which are publicly available. For the research study, we gained ethical approval to have a copy of the recording for research purposes only. When we completed analyses of the observations, the recordings were deleted.**

 It was unclear what your assumed response rate for the survey would be: “We aimed to receive a range of between 160 to 240 responses based on the average size of 20 committee members per research programme.“ Was this your aim or was this the maximum number of possible respondents? 20* how many programmes?

**Thank you for your comment. We have added “(a range of 8 to 12 research programmes included in the cohort).” to state the number of research programmes included in the cohort. Each funding committee can vary in size, therefore we took the average of 20 across the minimum and maximum number of committees.**

 Table 1: Will you explain what some of the barriers respondents considered might be? Or the scale of those barriers? Simply asking ‘are there barriers’ suggests that the answer is yes (because it is difficult to assert the complete absence of any barriers), but how intense are those barriers and what is the net effect with the positives?

**The barriers highlighted in the survey are presented in the manuscript under the relevant themes (and under Table 1), complementing the observations and interview data, rather than reporting on the survey responses as a single data source to the study.**

 I felt that the authors could expand upon why or how virtual meetings are “less free flowing and more structured” or face-to-face meetings less so. You say it’s clear they are conducted differently but don’t explain why. In face-to-face meetings to people talk over each other more? Have more whispered asides between participants? Why isn’t/shouldn’t a face-to-face meeting be “structured”?

**The focus of the study was not to compare to face-to-face as we did not observe any face-to-face meetings as part of the study (all committee meetings were virtual). The comment was made by an interviewee (and similar comments by others). It is possible that it felt more structured as there was no social element and time keeping due to not having run virtual committees before. We have amended the paragraph to accommodate your comment.**

 Typos / quick fixes

- The image resolution in Figure 1 and Figure 2 is poor making the text appear fuzzy and difficult to read.
- Either the authors have introduced a typo into the quote, “Some people do not like the longet silences and find it hard to cope with,” or the quote could benefit from a [sic].

**The images were provided to F1000 and we will look at the resolution to ensure it is if high quality (for both figures). We have amended figure 2 completely and have adjusted figure 1 resolution.**

**Thank you. This is a typo error and we have corrected it.**

# Reviewer report: Reviewer response for version 1

**DOI:** 10.5256/f1000research.159553.r284400

**Published:** 2024-06-21

**Authors:** Carter Bloch

**Affiliations:** 1 Aarhus University, Aarhus, Denmark

This is an interesting paper on a very relevant topic. It is important to understand how funding review processes work, in particular the use of online meetings to deliberate on proposals. The paper is well-written and clear. The paper is based on a mixed methods data collection which is very thoroughly described. The analysis is also very clear, where the paper brings out a number of interesting results, and utilizes a number of interview quotes and survey results to support the analysis. I have a number of more specific comments here:

 The “results” part of the abstract describes the three main themes of the coding analysis, but I question whether these are really results. I think the paper has a number of more concrete findings, which could be summarized here.

 The paper refers to whether the review processes are “effective, fair and transparent”. It would be helpful to spell out what is meant by these, particularly in the analysis where the paper concludes that online processes are “fair”. It would be good to elaborate on this (for example, in the Conclusion).

 For the ethical process, you note that a Participant Information Sheet was sent out to informants prior to the data collection, but you also secured written consent from all informants? Typically it is either one or the other.

 One aspect that I would have liked to see elaborated in greater detail is the issue of whether or how online meetings influence the scoring and selection process. Does it affect who receives the grants, and potentially also the diversity of projects (or other characteristics), or are there any indications of this? The overall conclusion appears to be that it does not affect the process, but the analysis hints at differences that could come into play in this regard. An example here could be in terms of how cases with the greatest difference in individual assessment are handled, or to what extent all committee member opinions are solicited and taken into consideration.

 Connected to this, it would be helpful to provide greater detail on the scoring process and how proposals are accepted.

 The analysis is based on three data sources, observation, interviews and a survey. Based on my read of the method and data collection, I expected that the observation study would be at the centre of the analysis, supplemented by insights from the interviews and the survey. However, my impression of the results section is that it is the other way around. There are a few places where the analysis draws on the observations, but they are few and far between. It would be helpful to bring the observations more into play in the analysis, particularly concerning discussions and selection of projects.

Is the work clearly and accurately presented and does it cite the current literature?

Yes

If applicable, is the statistical analysis and its interpretation appropriate?

Not applicable

Are all the source data underlying the results available to ensure full reproducibility?

No

Is the study design appropriate and is the work technically sound?

Yes

Are the conclusions drawn adequately supported by the results?

Yes

Are sufficient details of methods and analysis provided to allow replication by others?

No

Reviewer Expertise:

Research funding, research evaluation

I confirm that I have read this submission and believe that I have an appropriate level of expertise to confirm that it is of an acceptable scientific standard.

# Response

**Published:** 2024-07-02

**Authors:** Amanda Blatch-Jones

**Affiliations:** Faculty of Medicine, University of Southampton, Southampton, Hampshire, UK

The “results” part of the abstract describes the three main themes of the coding analysis, but I question whether these are really results. I think the paper has a number of more concrete findings, which could be summarized here.

**Thank you for your comment. Due to the word count it isn’t possible to add more to the results section, so we opted to include the identified themes along with the subthemes.**

 The paper refers to whether the review processes are “effective, fair and transparent”. It would be helpful to spell out what is meant by these, particularly in the analysis where the paper concludes that online processes are “fair”. It would be good to elaborate on this (for example, in the Conclusion).

**Thank you for your comment. We have amended the introduction to take account of your comment.**

 For the ethical process, you note that a Participant Information Sheet was sent out to informants prior to the data collection, but you also secured written consent from all informants? Typically it is either one or the other.

**The PIS was not used to secure participant consent, a separate process was used to gain consent from the participants. The PIS gave information about the study and why we were contacting them. Some participants completed the written consent form, but others did not. To be consistent and ensure that those who were interviewed were comfortable, the researcher read the consent form with the participants prior to conducting the interview (and gained verbal consent and give the participant the opportunity to ask questions prior to the interview).**

**For the survey, written consent was requested prior to completing the survey.**

**Written consent was not requested from the observations as consent was gained from the programme directors prior to the meeting and all members had a pre-emptive opt-out option two weeks prior to the meeting.**

 One aspect that I would have liked to see elaborated in greater detail is the issue of whether or how online meetings influence the scoring and selection process. Does it affect who receives the grants, and potentially also the diversity of projects (or other characteristics), or are there any indications of this? The overall conclusion appears to be that it does not affect the process, but the analysis hints at differences that could come into play in this regard. An example here could be in terms of how cases with the greatest difference in individual assessment are handled, or to what extent all committee member opinions are solicited and taken into consideration. Connected to this, it would be helpful to provide greater detail on the scoring process and how proposals are accepted.

**Thank you for your comment. The scoring process did not differ to what was used in face-to-face meetings prior to COVID-19. Therefore, the main focus was not on the differences in the scoring but whether there were any practical challenges through doing this online. We have described some of these differences but we found that it did not affect the virtual process for decision-making.** **We have now added information about the scoring process, which also picks up your point about how proposals are accepted (e.g., proposals are ranked at the end to determine which applications meet the threshold).**

 The analysis is based on three data sources, observation, interviews and a survey. Based on my read of the method and data collection, I expected that the observation study would be at the centre of the analysis, supplemented by insights from the interviews and the survey. However, my impression of the results section is that it is the other way around. There are a few places where the analysis draws on the observations, but they are few and far between. It would be helpful to bring the observations more into play in the analysis, particularly concerning discussions and selection of projects.

**We have tried to be consistent on drawing from all three data sources, given the richness of the data and length of the manuscript. The development of the themes and sub-themes were predominantly from the observations with the survey and interview data used to confirm what was observed from the perspective of the participants.** **The quotes used in the manuscript come mainly from the survey and interviews purely because conversations were not recorded verbatim during the observations.  The quotes are used to add context to the data but the observations played a big role in the development of the themes. As directed by the research questions, we explored the role of virtual funding committees to inform decision-making, rather than focusing on how applications were selected. We feel that the discussions during the committee meetings are consistently covered in the results, which forms the basis of the themes.**

# Reviewer report: Reviewer response for version 1

**DOI:** 10.5256/f1000research.159553.r284406

**Published:** 2024-06-03

**Authors:** Adrian Barnett

**Affiliations:** 1 Queensland University of Technology, Brisbane, Queensland, Australia

This is an interesting and worthwhile study to examine panel dynamics using a real process. There was a good balance of data collection from reviewers and staff. It appears to be well planned and well conducted. The findings are likely useful for other funders.

 Perhaps it is out of scope, but there is some debate on whether panels can be removed in favour of independent peer reviewers. As well as the time and money costs of panels, there are concerns that they reduce the variance in scores towards strong voices [1], and that committee members inevitably score proposals which are outside their expertise. Some studies have found that panels are not be useful for decision making [2,3].

 One anecdotally cited benefit of panels is that they provide extra scrutiny, as reviewers cannot be disengaged, e.g. https://x.com/GillBedi/status/1697876646936928333. Were there any comments on this?

 "noisy voices are heard more" – this is the same in a face-to-face meeting: "some of the more dominant personalities to take over the group and have an undue influence on the grant outcomes" [1].

 "the larger the committee the more opportunities for bias could be introduced", but too small can be seen to not have enough voices [4]. There is an interesting balance to strike here.

 Having "networking opportunities" is in my opinion a negative aspect of panels. It provides an advantage to those who are invited and can attend the meeting. It potentially contributes the Matthew effect in research funding [5], as those who attend the meeting get privileged insights into how to write their next application.

 Minor comments

 - PIS = participant information sheet?

 - "This study conducted according to the principles" missing 'was'

 - Pie charts are not a recommended data visualisation, see for example https://scc.ms.unimelb.edu.au/resources/data-visualisation-and-exploration/no_pie-charts

 - A consent rate of 6 out of 10 committees is disappointing given the aim to improve the process. I realise this was beyond the researchers' control.

 - The questions shown in Table 1 have a positive slant towards the online format. More neutral wording would have been preferable.

 -“Earlier proposals in the day received wider participation but by the end of the meeting, few people could contribute, understandable as it was a long day.” – this is likely the same in face-to-face meetings.

 - A description of the scoring process may be useful background.

 - "view an unconscious bias video prior to reviewing applications" the NHMRC encourage reviewers to do the unconscious bias training (“Harvard Implicit Bias” https://www.nhmrc.gov.au/funding/peer-review).

 - "some clustering of scores" clustering within the scores from the same committee member (e.g., scores for feasibility, novelty) or across the committee?

 - Typo: "Helsinkiv".

Is the work clearly and accurately presented and does it cite the current literature?

Partly

If applicable, is the statistical analysis and its interpretation appropriate?

Not applicable

Are all the source data underlying the results available to ensure full reproducibility?

No

Is the study design appropriate and is the work technically sound?

Yes

Are the conclusions drawn adequately supported by the results?

Yes

Are sufficient details of methods and analysis provided to allow replication by others?

Yes

Reviewer Expertise:

research funding, statistics

I confirm that I have read this submission and believe that I have an appropriate level of expertise to confirm that it is of an acceptable scientific standard.

## References

[1] : 'Are you siding with a personality or the grant proposal?': observations on how peer review panels function. *Res Integr Peer Rev* .2017;2: 10.1186/s41073-017-0043-x 19 10.1186/s41073-017-0043-x 29451548 PMC5803633

[2] : Panel discussion does not improve reliability of peer review for medical research grant proposals. *J Clin Epidemiol* .2012;65(1) : 10.1016/j.jclinepi.2011.05.001 47-52 10.1016/j.jclinepi.2011.05.001 21831594

[3] : Using simplified peer review processes to fund research: a prospective study. *BMJ Open* .2015;5(7) : 10.1136/bmjopen-2015-008380 e008380 10.1136/bmjopen-2015-008380 26137884 PMC4499682

[4] : Evaluation of research proposals by peer review panels: broader panels for broader assessments?. *Science and Public Policy* .2023;50(4) : 10.1093/scipol/scad009 619-632 10.1093/scipol/scad009

[5] : The Matthew effect in science funding. *Proc Natl Acad Sci U S A* .2018;115(19) : 10.1073/pnas.1719557115 4887-4890 10.1073/pnas.1719557115 29686094 PMC5948972

# Response

**Published:** 2024-07-02

**Authors:** Amanda Blatch-Jones

**Affiliations:** Faculty of Medicine, University of Southampton, Southampton, Hampshire, UK

Perhaps it is out of scope, but there is some debate on whether panels can be removed in favour of independent peer reviewers. As well as the time and money costs of panels, there are concerns that they reduce the variance in scores towards strong voices [1], and that committee members inevitably score proposals which are outside their expertise. Some studies have found that panels are not be useful for decision making [2,3].

**Thank you for the additional references you have cited.**

 One anecdotally cited benefit of panels is that they provide extra scrutiny, as reviewers cannot be disengaged, e.g. https://x.com/GillBedi/status/1697876646936928333. Were there any comments on this?

**Our findings indicated that the committee engaged in good quality discussion with input from multiple members indicating that members were engaged with the review process.  In addition, data not reported in this paper demonstrate that committee members felt that the reviewing, critically appraising and discussing applications was the most cited role of committee members.  However, as noted in the paper, engagement throughout the whole meeting was sometimes difficult to gauge as cameras were not always on and members felt there was the potential to be more distracted on virtual calls.**

 "noisy voices are heard more" – this is the same in a face-to-face meeting: "some of the more dominant personalities to take over the group and have an undue influence on the grant outcomes" [1].

**We have added this as an additional example of similarities in face to face and virtual meetings in the discussion (pg. 24)**

 "the larger the committee the more opportunities for bias could be introduced", but too small can be seen to not have enough voices [4]. There is an interesting balance to strike here.

**Thank you for your thoughtful comment, and it is an important point to raise. Our paper reported how we found smaller committees to be more informal than larger committees.  We agree that there is a balance to strike, and although there has been some research into ideal numbers of peer reviewers and committee members there is no consensus.  Future work could explore this in more detail**

 Having "networking opportunities" is in my opinion a negative aspect of panels. It provides an advantage to those who are invited and can attend the meeting. It potentially contributes the Matthew effect in research funding [5], as those who attend the meeting get privileged insights into how to write their next application.

**We have adjusted the point raised in the discussion to highlight the potential advantage of being a committee member, although also highlighting that virtual meetings could be a way to facilitate the diversity of the committee.**

 Minor comments

 - PIS = participant information sheet?

 - "This study conducted according to the principles" missing 'was'

 - Pie charts are not a recommended data visualisation, see for example https://scc.ms.unimelb.edu.au/resources/data-visualisation-and-exploration/no_pie-charts

**We have changed the pie chart to a more suitable diagram.**

 - A consent rate of 6 out of 10 committees is disappointing given the aim to improve the process. I realise this was beyond the researchers' control.

**As you point out the consent rate was unfortunately out of our control. However, having included two programmes with more than one committee provided us with the opportunity to explore changes over time, which is a unique feature to the study.**

 - The questions shown in Table 1 have a positive slant towards the online format. More neutral wording would have been preferable.

**We understand that the questions from the survey appear to be more directional to virtual meetings. However, the purpose of the survey was to understand committee members’ viewpoint on virtual rather than compared to face-to-face.**

 -“Earlier proposals in the day received wider participation but by the end of the meeting, few people could contribute, understandable as it was a long day.” – this is likely the same in face-to-face meetings.

 - A description of the scoring process may be useful background.

**We have provided additional information about the scoring on page 15.**

 - "view an unconscious bias video prior to reviewing applications" the NHMRC encourage reviewers to do the unconscious bias training (“Harvard Implicit Bias” https://www.nhmrc.gov.au/funding/peer-review).

**Thank you. We have included the NHMRC as an example of a funding organisation actively encouraging reviewers to do unconscious bias training.**

 - "some clustering of scores" clustering within the scores from the same committee member (e.g., scores for feasibility, novelty) or across the committee?

**We have amended the clustering of scores sentence to provide greater clarity on this point.**

 - Typo: "Helsinkiv".

**Thank you for your comments. We have addressed the typos accordingly and considered your additional comments in revising the manuscript.**
